# Association Between Sleep and Physical Function in a Longitudinal TLC Cohort Study of Women

**DOI:** 10.1093/geroni/igaf122.490

**Published:** 2025-12-31

**Authors:** Cynthia Kusters, Wanting Zhai, Jeanne Mandelblatt, Judith Carroll

**Affiliations:** University of California, Los Angeles, Los Angeles, California, United States; Georgetown University, Washington, District of Columbia, United States; Georgetown University, Washington, District of Columbia, United States; University of California, Los Angeles, Los Angeles, California, United States

## Abstract

Sleep disturbances, such as insomnia, are common in postmenopausal women and are believed to negatively affect physical functioning. These disturbances are more prevalent in breast cancer survivors compared to the general female population. However, limited data exists on how sleep affects physical function in older breast cancer cases. We analyzed data from the Thinking and Living with Cancer study, a multicenter, longitudinal study of women aged 60+ newly diagnosed with breast cancer and frequency-matched non-cancer controls. Participants were followed annually for up to 5-years. Sleep quality was assessed using the PSQI (score >5 indicating sleep disruption); physical functioning was measured by the SF-12 physical components score (PCS) and the Timed Up and Go (TUG) test. Linear mixed models analyzed 2,659 visits from 771 women (341 controls, 430 survivors), adjusting for age, race, comorbidities, recruitment site, and visit time. The average age of the sample was 69.5 (70.0 vs. 69.3 in survivors vs. controls). Disrupted sleep was more prevalent among survivors at baseline (43.7% vs. 31.1%;p<0.001) and persisted during follow-up. Compared to controls without sleep disruption, survivors with sleep disturbances had ∼0.47 SD lower adjusted PCS scores (p < 0.001). Survivors with sleep disruption also had longer TUG times (∼1.3 sec) than controls without sleep disturbances, especially in the first 24 months (p = 0.002). These findings suggest older women with breast cancer and sleep disruption are vulnerable to long-term losses in physical functioning. Clinical surveillance for sleep disruption paired with intervention could help maintain function in the growing population of older cancer survivors.

